# Etiological Subgroups of Small-for-Gestational-Age: Differential Neurodevelopmental Outcomes

**DOI:** 10.1371/journal.pone.0160677

**Published:** 2016-08-08

**Authors:** Xiuhong Li, Rina D. Eiden, Leonard H. Epstein, Edmond D. Shenassa, Chuanbo Xie, Xiaozhong Wen

**Affiliations:** 1 Department of Maternal and Child Health, School of Public Health, Sun Yat-sen University, Guangzhou, Guangdong 510080, China; 2 Division of Behavioral Medicine, Department of Pediatrics, University at Buffalo, State University of New York, Buffalo, NY, 14214, United States of America; 3 Research Institute on Addictions, University at Buffalo, State University of New York, Buffalo, NY, 14203, United States of America; 4 Maternal and Child Health Program, School of Public Health, University of Maryland, College Park, MD, 20742, United States of America; 5 Department of Epidemiology and Biostatistics, School of Public Health, University of Maryland, College Park, MD, 20742, United States of America; 6 Department of Epidemiology, School of Public Health, Brown University, Providence, RI, 02912, United States of America; 7 Department of Cancer Prevention Research, Sun Yat-sen University Cancer Center, Guangzhou, Guangdong Province 510060, China; Universite de Montreal, CANADA

## Abstract

**Objectives:**

It remains unclear why substantial variations in neurodevelopmental outcomes exist within small-for-gestational-age (SGA) children. We prospectively compared 5-y neurodevelopmental outcomes across SGA etiological subgroups.

**Methods:**

Children born SGA (N = 1050) from U.S. Early Childhood Longitudinal Study-Birth Cohort (2001–2007) was divided into etiological subgroups by each of 7 well-established prenatal risk factors. We fit linear regression models to compare 5-y reading, math, gross motor and fine motor scores across SGA subgroups, adjusting for socio-demographic confounders.

**Results:**

Compared to singleton SGA subgroup, multiple-birth SGA subgroup had lower mean reading (adjusted mean difference, -4.08 [95% confidence interval, -6.10, -2.06]) and math (-2.22 [-3.61, -0.84]) scores. These disadvantages in reading and math existed only among multiple-birth SGA subgroup without ovulation stimulation (reading, -4.50 [-6.64, -2.36]; math, -2.91 [-4.37, -1.44]), but not among those with ovulation stimulation (reading, -2.33 [-6.24, 1.57]; math 0.63 [-1.86, 3.12]). Compared to singleton SGA subgroup without maternal smoking and inadequate gestational weight gain, singleton SGA subgroup with co-occurrence of maternal smoking and inadequate gestational weight gain (GWG) had lower mean reading (-4.81 [-8.50, -1.12]) and math (-2.95 [-5.51, -0.38]) scores. These differences were not mediated by Apgar score.

**Conclusions:**

Multiple-birth SGA subgroups (vs. singleton SGA) or singleton SGA subgroup with co-occurrence of smoking and inadequate GWG (vs. singleton SGA subgroup without maternal smoking and inadequate gestational weight gain) have poorer cognitive development up to 5 y.

## Introduction

Body size at birth is well known to be a risk factor for perinatal morbidity and mortality, as well as poor neurodevelopment [1,2]. A newborn shorter or lighter than expected for a certain gestational age, i.e. 2 standard deviation (SD) below the average or below the 3^rd^ or 10^th^ percentile of newborn reference of the same sex and gestational age is often called small for gestational age (SGA). About 10% -16% of children in high-income countries [3] and 27% of children in low- and middle-income countries [4] are born small-for-gestational-age (SGA), due to various genetic and environmental causes. Some key prenatal risk factors for SGA include maternal pre-pregnancy underweight, short stature, smoking during pregnancy, alcohol use during pregnancy, inadequate gestational weight gain (GWG), hypertensive conditions, and multiple births [5].

On average, children born SGA are more likely to have worse neurodevelopment than children born appropriate-for-gestational-age (AGA) [6]. However, there is a great deal of heterogeneity in these adverse long-term outcomes among SGA newborns. It remains unclear why substantial variations in long-term neurodevelopmental outcomes exist within small-for-gestational-age (SGA) children. Neurodevelopmental disadvantages related to SGA are likely due to structural and/or functional impairment in brain development [7]. Research suggests that different risk factors of SGA may influence fetal brain development through different biological mechanisms. For example, SGA fetus with maternal smoking during pregnancy may have interferes directly by stimulation of neurotransmitters or indirectly by inducing brain hypoxia [8,9]; SGA fetus with maternal pre-pregnancy underweight or inadequate GWG may have insufficient supply of nutrition to brain during pregnancy [10,11]; SGA fetus with multiple birth often restricted by crowded space and nutrition supply [12,13]. In addition, some prenatal risk factors leading to SGA are likely to co-occur, which may cause “double hits” to the fetal brain and causes more severe impairments than each factor separately. Therefore, it’s reasonable to hypothesize different etiologies of SGA can help to understand these substantial variations. This hypothesis is supported by our previous research in which only SGA subgroups with maternal smoking during pregnancy have elevated risk of hypercholesterolemia in adults, while SGA subgroups without maternal smoking have similar risk as AGA children [14]. The death risk of a SGA fetus is higher if the mother is ≥175 cm tall, normotensive, nulliparous, or non-smoking [15].

**Fig 1:**
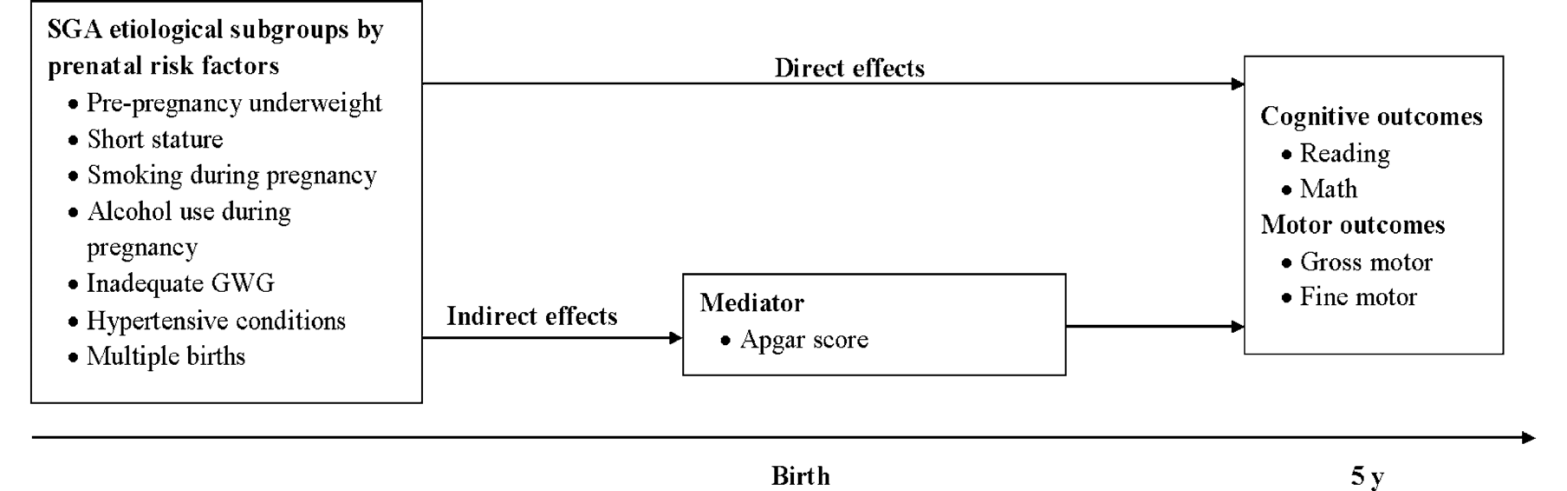
Life course framework for neurodevelopmental outcomes at 5 y of SGA etiological subgroups.

In addition, the extent of neurodevelopmental impairment may vary considerably across SGA etiological subgroups, possibly due to the variation in Apgar score (an indicator for some SGA-related complications such as birth asphyxia and cerebral palsy [16]). This variable is associated with the incidence and severity of late neurodevelopmental impairment, possibly through reduction in brain weight and cell number [17]. Alternatively, the variation in neurological impairment across SGA subgroups may be due to the direct effects of some prenatal risk factors on the developing fetal brain. As shown in **Fig 1**, we hypothesized that etiological subgroups of SGA have different neurodevelopment measured by cognitive and motor outcomes, through the direct effects of SGA-related prenatal risk factors or mediation by Apgar score (indirect effects).

Early screening and intervention may alter SGA children’s disadvantaged neurodevelopmental paths and thus improve their later outcomes [18–20]. Dividing SGA children into etiological subgroups, can not only help to distinguish the perinatal etiology for SGA, but also identify high-risk subgroups of SGA that have poorer neurodevelopmental outcomes in later life and thus need early intervention. Therefore, in this study we aimed to 1) examine the differences in neurodevelopmental outcomes at 5 y across etiological subgroups of SGA based on 7 key prenatal risk factors including maternal pre-pregnancy underweight, short stature, smoking during pregnancy, alcohol use during pregnancy, inadequate gestational weight gain (GWG), hypertensive conditions, and multiple births; 2) examine the differences in neurodevelopmental outcomes at 5 y across etiological subgroups of SGA based on co-occurrence of significant risk factors selected in Aim 1; 3) examine the extent to which Apgar score would mediate the associations between SGA etiological subgroups and neurodevelopmental outcomes.

## Materials and Methods

### Data and sample

**Fig 2:**
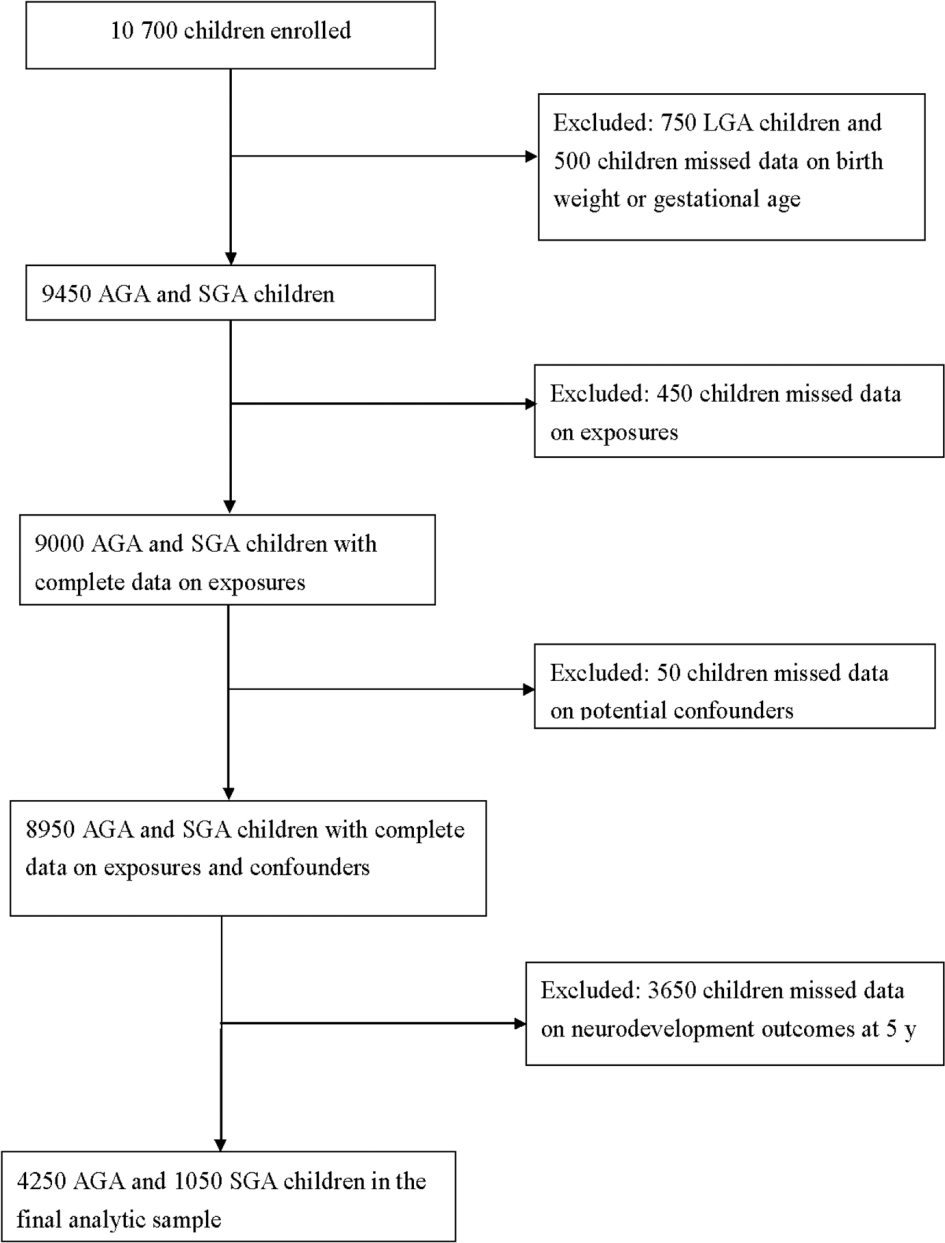
Flow chart of the analytic sample.

We used existing data from the Early Childhood Longitudinal Study-Birth cohort (ECLS-B, 2001–2007, N = 10 700), a U.S. national longitudinal birth cohort with child assessments at birth, 9 months (m), 2 years (y), 4 y (preschool), 5 y (kindergarten 2006), and 6 y (kindergarten 2007) [21,22]. This analysis included ECLS-B children born SGA (N = 1050) and AGA (N = 4250) with complete data on birth weight and gestational age, the 7 key prenatal risk factors for SGA, neurodevelopmental outcomes at 5 y, and potential confounders (**Table 1**). **Fig 2** shows the sample flow. All reported numbers about sample size are rounded to nearest 50 according to the confidentiality policy of U.S. Department of Education. This secondary data analysis was approved by the Social and Behavioral Sciences Institutional Review Board, State University of New York at Buffalo.

**Table 1:** Characteristics of analytic and excluded SGA and AGA samples (N = 9450).

	Analytic SGA and AGA sample (N = 5300)		Excluded SGA and AGA sample (N = 4150)^a^		
Characteristics	N(%)	mean(SD)	N (%)	mean(SD)	*P-value*^*b*^
**HOUSEHOLD**					
Family socioeconomic status		0.0 (0.9)		-0.2 (0.8)	**<0.001**
**MOTHER**					
Age at pregnancy		27.8 (6.4)		27.0 (6.4)	**<0.001**
Race					
Non-Hispanic white	2500 (47.4)		1950 (47.6)		
Non-Hispanic black	950 (18.0)		750 (17.5)		
Hispanics	800 (14.7)		750 (18.1)		**<0.001**
Asian/Pacific Islander	800 (14.9)		550 (13.7)		
American Indian	250 (5.0)		150 (3.1)		
Education level					
Below high school	900 (16.6)		950 (22.5)		
High school or equivalent	1550 (29.2)		1350 (33.0)		**<0.001**
College	2200 (42.2)		1500 (35.7)		
Graduate or above	650 (12.1)		350 (8.8)		
Married	3650 (68.7)		2600 (62.2)		**<0.001**
Vaginal delivery	3500 (66.5)		2750 (66.5)		0.956
Diabetes during pregnancy	200 (3.4)		150 (3.5)		0.751
Pre-pregnancy underweight (BMI<18.5 kg/m^2^)	350 (6.4)		350 (8.0)		**0.004**
Pre-pregnancy short stature (height<157.5 cm)	1450 (27.2)		1150 (28.1)		0.310
Smoking status during pregnancy					
Never	4150 (78.1)		3100 (74.2)		**<0.001**
Quitted	450 (9.1)		450 (10.3)		
Moderate smoking (1–9 cigs/day)	350 (6.3)		350 (8.0)		
Heavy smoking (10+ cigs/day)	350 (6.6)		300 (7.5)		
Alcohol use during pregnancy	200 (3.5)		150 (3.2)		0.417
Inadequate GWG	1750 (33.0)		1400 (33.3)		0.747
Hypertensive conditions	400 (7.5)		300 (6.9)		0.256
Multiple Births	1050 (19.7)		600 (14.4)		**<0.001**
Without ovulation stimulation	800 (15.4)		500 (12.4)		
With ovulation stimulation	250 (4.3)		100 (2.4)		
**CHILD**					
Male sex	2650 (49.7)		2200 (52.7)		**0.004**
Gestational age, weeks		37.5 (3.6)		37.1 (4.1)	**<0.001**
Preterm birth	1250 (26.4)		1200 (29.1)		**0.004**
Birth weight, gram		2875.3 (798.4)		**2801.3 (876.0)**	**<0.001**
Birth-weight-for-gestational-age percentile		37.3 (26.4)		37.5 (27.0)	0.764

SD, standard deviation; BMI, body mass index; GWG, gestational weight gain.

Significant results are bolded

^a^ Chi-square test for categorical variables and *t*-test for continuous variables.

^tb^ The sum of categories of some characteristics may be less than 100% due to missing data.

### Measures of exposures

In this study, the exposures were SGA etiological subgroups based on each or co-occurrence of maternal pre-pregnancy underweight, short stature, smoking and alcohol use during pregnancy, inadequate GWG, hypertensive conditions, and multiple births. We considered these 7 factors because they are well-established prenatal risk factors for SGA and could explain large proportion of SGA [5]. Also, they are potentially modifiable by nutritional, behavioral, and medical interventions.

#### SGA and AGA

Birth weight in grams, last menstrual period (LMP), and delivery date were extracted from birth certificates of live births in 2001 provided by the State Vital Registration and Statistics Executives [23]. Birth weight in birth certificate has been shown valid and reliable (e.g. correlation with birth weight on birth record, 0.96) [24]. Gestational age in weeks was calculated as the interval between LMP and delivery date. Gestational age from birth certificate has been shown fairly valid (e.g. correlation with gestational age on birth record, 0.68) [24]. SGA was defined as birth weight below 10^th^ percentile for the same sex and gestational age within a national reference population of U.S. newborns [25]. AGA was defined as birth weight between 10^th^-90^th^ percentiles. For the purpose of this analysis, we excluded children with birth weight above 90^th^ percentile (large-for-gestational-age, LGA).

#### Pre-pregnancy underweight and short stature

Mother reported her height and pre-pregnancy weight in the 9-m postpartum interview. Pre-pregnancy body mass index (BMI) was calculated as weight (kg)/height (m) ^2^. Pre-pregnancy underweight was defined as pre-pregnancy BMI<18.5kg/m^2^ [26]. Short stature was defined as maternal height ≤157.5cm [27].

#### Smoking and alcohol use during pregnancy

We extracted information on maternal smoking (# of cigarettes/day) and alcohol use (yes vs. no) during pregnancy from birth certificates. Although these two socially unfavorable behaviors were subject to under-report in birth certificate, it was acceptable to use them for exploratory studies like ECLS-B [28]. If the information on smoking (16.1%) and alcohol use (14.9%) was unavailable in birth certificate, we supplemented it with retrospective self-report in the 9-m postpartum interview. Besides binary variable (yes/no), we also classified maternal smoking into 4 categories: never smoking, quitted smoking (smoking before pregnancy, but not during pregnancy), moderate smoking (1–9 cigarettes/day during pregnancy) and heavy smoking (≥10 cigarettes/day during pregnancy).

#### Inadequate GWG

The information on total GWG was obtained from birth certificates (77.9%) and also from maternal self-reports at 9-m postpartum interview (22.1%) if birth certificate data was unavailable. Inadequate GWG for singletons was defined total GWG less than 12.5 kg for underweight (pre-pregnancy BMI<18.5 kg/m^2^), 11.5 kg for normal weight (BMI, 18.5–24.9 kg/m^2^), 7 kg for overweight (BMI, 25–29.9 kg/m^2^), and 5 kg for obese women (BMI ≥30 kg/m^2^), respectively. For multiple births, inadequate GWG was defined total GWG less than 17 kg for underweight and normal weight, 14 kg for overweight, and 11 kg for obese women, respectively [26].

#### Hypertensive conditions

The information on diagnoses of hypertensive conditions (i.e., chronic hypertension, gestational hypertension, preeclampsia, and eclampsia) was extracted from birth certificates.

#### Multiple births

The information on singleton and multiple births (e.g., twins and triplets) was extracted from birth certificates. Ovulation stimulation is one of the most important reasons for recent rapid increase in rate of multiple births [29]. Thus we further divided multiple births into those with or without ovulation stimulation based on maternal retrospective recall at 9-m postpartum interview.

### Measures of outcomes

Our key neurodevelopmental outcome measures included reading, math, gross and fine motor scores at 5 y assessed by certified fieldworkers (**Table 2**). Neurodevelopmental status assessed around the age for kindergarten has been shown to be strongly related to later school success up to high school [30]. Although longer-term follow-up was preferred, we decided not to use 6-y outcome data, as the 6-y ECLS-B sample was rather small (<20% of full sample) and highly selective [22].

**Table 2:** Comparison of 5-y neurodevelopmental outcomes between children born SGA and AGA (N = 5300).

	Mean (SD)			*P*-value in t-test	Adjusted mean difference (95% CI), SGA-AGA^a^
	Total	SGA (N = 1050)	AGA (N = 4250)		
**Cognitive outcomes**					
Reading	40.25 (15.50)	38.19 (15.04)	40.76 (15.58)	**<0.001**	**-1.05 (-1.93, -0.16)**
Math	41.21 (10.72)	39.49 (10.71)	41.64 (10.68)	**<0.001**	**-0.84 (-1.45, -0.22)**
**Motor outcomes**					
Gross motor	4.70 (1.69)	4.50 (1.76)	4.76 (1.68)	**<0.001**	**-0.22 (-0.34, -0.10)**
Fine motor	3.18 (1.48)	2.92 (1.59)	3.25 (1.45)	**<0.001**	**-0.22 (-0.32, -0.12)**

SGA, small-for-gestational-age; AGA, appropriate-for-gestational-age; SD, standard deviation; CI, confidence interval.

Gross motor score range, 0–7; Fine motor score range, 0–5.

Significant results are bolded.

^a^ Adjusted for family socioeconomic status; maternal age at pregnancy, race/ethnicity, educational level, marital status, method of delivery, and diabetes during pregnancy; and child’s sex.

#### Reading and math

The assessment battery of reading and math in ECLS-B kindergarten wave was a combination of some items fielded as part of the ECLS-B preschool wave and some items used with kindergartners in another cohort, the Early Childhood Longitudinal Study, Kindergarten Class of 1998–99 (ECLS-K) battery. Specifically, the 5-y reading assessment was based on the child’s performance on certain language-based items (receptive language/ Peabody Picture Vocabulary Test items) and the literacy items (e.g., conventions of print, letter recognition, understanding of letter-sound relationships, phonological awareness, sight word recognition, understanding words in the context of simple sentences). The 5-y math assessment included number sense, properties, operations, measurement, geometry and spatial sense, data analysis, statistics, probability, patterns, algebra, and functions. For reading and math, overall scale scores based on the full set of assessment items were calculated using item response theory (IRT) procedures [21]. IRT methods make it possible to calculate scores for a given domain that can be compared regardless of which version of the test battery a child received through adaptive testing. The mean±SD of IRT scale scores for reading and math were 38.60±14.84, 40.40±10.56, respectively. The mean±SE of IRT Theta ability estimates for reading and math were 0.33±0.34, 0.38±0.34, respectively. No floor or ceiling effects were found and reliability coefficients of IRT-based score were 0.92 for both the reading and math assessments in the kindergarten 2006 wave of ECLS-B [21,22]. No specific external validation for reading and math items was conducted in ECLS-B kindergarten 2006 sample, as these items had already validated in ECLS-B preschool wave and also ECLS-K kindergarten wave. For example, the ECLS-B preschool field test item pools were validated by concurrent administration of one of the six School Readiness subtests (i.e., colors, letters, numbers/counting, sizes, comparisons, and shapes) of the Bracken Basic Concept Scale-Revised [31]. The correlation of the Bracken Letters subtest with early reading of ECLS-B preschoolers was 0.82, while the correlation of the Bracken Numbers subtest mathematics field test ability estimate of ECLS-B preschoolers was 0.75.

#### Gross and fine motor

The gross and fine motor skills were direct assessed using a battery mainly modified from the preschool and/or kindergarten versions of the Early Screening Inventory-Revised (ESI-R) [21], a reliable and valid instrument to evaluate a child’s ability to perform developmentally appropriate tasks [32,33]. Gross motor skills were assessed by certified field workers using 7 physical tasks including balancing (left and right feet), hopping (left and right feet), skipping, walking backwards, and bean bag catching, which were standardized in the Child Assessment Booklet. For the bean bag catching task, if the child caught the bag all the five times, we rated the task as “pass”, otherwise as “fail”. For the other 6 tasks, fieldworkers rated each of them as either pass or fail. We calculated the total number of passed physical tasks as the gross motor score (range, 0–7). Fine motor skill tests included “build a gate” test with blocks and copying 4 forms (square, triangle, asterisk and circle-square) [21]. “Build a gate” task was scored by fieldworker, and the drawing tasks were scored by specially trained coders centrally at RTI International. All items were scored as a “pass” (1 point) or “fail” (0 point) using standardized scoring rules from the ESI-R, kindergarten version. We calculated the total number of passed items and used it as the fine motor score (range, 0–5). The coding of fine motor items was rather reliable, as both inter-rater and standard-comparison agreements were above 85% [21].

Note that child intelligence quotient (IQ) assessment was unavailable in ECLS-B due to the study priority on the learning-related skills (i.e., reading and math) that were more directly related to school readiness, intent to measure cognitive dimensions that were comparable across different assessment waves at different ages (2 y, 4 y, 5 y, and 6 y), as well as several important constraints including short assessment time (<45 minutes), less controlled home settings, and less professional field examiners with only basic knowledge of child development [21].

### Measures of mediators

We considered 5- minute Apgar score as a perinatal mediator related to poor neurodevelopment of some SGA etiological subgroups. Although 5- minute Apgar score is not designed to predict long term health and development, it can be a valuable indicator for some important fetal complications such as birth asphyxia and cerebral palsy [16].

### Measures of confounders

The confounders in this analysis included family socioeconomic status (SES); maternal age at pregnancy, race/ethnicity, education level, marital status, method of delivery, and diabetes during pregnancy; and the child’s sex. Family SES score was derived from 5 available components including parents’ education, parents’ occupation, and household income [23].

### Statistical analysis

In this study, we divided SGA children into etiological subgroup because it can better control for other prenatal risk factors than regular statistical methods such as multivariable regression modeling and thus offer clear separation of different etiologies of SGA. Although the subgroup approach is subject to lower statistical power due to reduced sample size, it fits our study purposes well.

#### Aim 1—single factor

We fitted multivariable linear regression models to compare neurodevelopmental outcomes between the etiological SGA subgroup with a specific risk factor and the SGA subgroup without the corresponding risk factor (SGA internal reference), adjusting for confounders (**Table 3**). All regression models were fitted with generalized estimating equations to control for the correlation between multiple siblings (twins or triplets), by specifying exchangeable covariance matrix among siblings.

**Table 3:** Neurodevelopmental outcomes at 5 y of SGA etiological subgroups by single prenatal risk factor (N = 1050).

		Adjusted mean difference in 5-y outcomes (95% CI)^a^			
		Cognitive outcomes		Motor outcomes	
	N (%)	Reading	Math	Gross	Fine
**By maternal pre-pregnancy underweight**					
SGA without underweight	950 (92.7)	Reference	Reference	Reference	Reference
SGA with underweight	100 (7.3)	-0.35 (-3.55, 2.84)	0.55 (-1.68, 2.79)	-0.12 (-0.48, 0.24)	0.17 (-0.14, 0.48)
**By maternal short stature**					
SGA without short stature	700 (66.4)	Reference	Reference	Reference	Reference
SGA with short stature	350 (33.6)	0.10 (-1.74, 1.94)	0.31 (-0.98, 1.59)	**0.34 (0.11, 0.58)**	0.07 (-0.14, 0.27)
**By maternal smoking during pregnancy**					
SGA without smoking	750 (71.7)	Reference	Reference	Reference	Reference
SGA with quitted smoking	100 (8.1)	-0.44 (-3.78, 2.91)	-0.85 (-3.24, 1.54)	0.15 (-0.30, 0.59)	-0.04 (-0.45, 0.37)
SGA with moderate smoking	100 (9.6)	-0.49 (-3.14, 2.16)	0.08 (-1.90, 2.06)	0.17 (-0.22, 0.55)	0.06 (-0.26, 0.38)
SGA with heavy smoking	100 (10.6)	-1.47 (-4.65, 1.70)	-1.64 (-3.89, 0.61)	-0.21 (-0.63, 0.21)	-0.07 (-0.40, 0.27)
**By maternal alcohol use during pregnancy**					
SGA without alcohol use	1000 (95.2)	Reference	Reference	Reference	Reference
SGA with alcohol use	50 (4.8)	-1.19 (-5.01, 2.64)	0.33 (-2.33, 2.99)	0.26 (-0.19, 0.71)	0.11 (-0.35, 0.58)
**By maternal GWG**					
SGA with normal GWG	600 (57.6)	Reference	Reference	Reference	Reference
SGA with inadequate GWG	450 (42.4)	**-2.26 (-3.98, -0.55)**	**-1.54 (-2.74, -0.35)**	-0.04 (-0.26, 0.18)	-0.19 (-0.38, 0.00)
**By maternal hypertensive conditions**					
SGA without hypertensive conditions	950 (88.1)	Reference	Reference	Reference	Reference
SGA with hypertensive conditions	100 (11.9)	0.29 (-2.37, 2.96)	0.28 (-1.52, 2.08)	-0.20 (-0.55, 0.15)	-0.10 (-0.39, 0.20)
**By multiple births**					
Singleton SGA	650 (65.6)	Reference	Reference	Reference	Reference
Multiple-birth SGA	350 (34.4)	**-4.08 (-6.10, -2.06)**	**-2.22 (-3.61, -0.84)**	0.20 (-0.07, 0.47)	-0.11 (-0.35, 0.13)
Without ovulation stimulation	250 (26.8)	**-4.50 (-6.64, -2.36)**	**-2.91 (-4.37, -1.44)**	0.21 (-0.07, 0.50)	-0.12 (-0.37, 0.14)
With ovulation stimulation	100 (7.6)	-2.33 (-6.24, 1.57)	0.63 (-1.86, 3.12)	0.13 (-0.37, 0.64)	-0.09 (-0.47, 0.30)

SGA, small-for-gestational-age; AGA, appropriate-for-gestational-age; GWG, gestational weight gain; CI, confidence interval.

Gross motor score range, 0–7; Fine motor score range, 0–5.

Significant results are bolded.

Definitions of prenatal risk factors:

Pre-pregnancy underweight: BMI<18.5kg/m^2^;

Maternal short stature: height ≤157.5cm;

Smoking: never smoking, quitted smoking (smoking before pregnancy, but not during pregnancy), moderate smoking (1–9 cigarettes/day during pregnancy) and heavy smoking (≥10 cigarettes/day during pregnancy)

Inadequate GWG: for singletons, total GWG less than 12.5 kg for underweight (pre-pregnancy BMI<18.5 kg/m2), 11.5 kg for normal weight (BMI, 18.5–24.9 kg/m2), 7 kg for overweight (BMI, 25–29.9 kg/m2), and 5 kg for obese women (BMI ≥30 kg/m2), respectively. For multiple births, total GWG less than 17 kg for underweight and normal weight, 14 kg for overweight, and 11 kg for obese women, respectively.

Hypertensive conditions: chronic hypertension, gestational hypertension, preeclampsia, and eclampsia

Multiple births: twins and triplets.

^a^ Adjusted for family socioeconomic status; maternal age at pregnancy, race/ethnicity, educational level, marital status, method of delivery, and diabetes during pregnancy; and child’s sex.

#### Aim 2- co-occurring factors

Restricted by sample size, we only considered the co-occurrence of 3 relatively common risk factors (i.e., smoking during pregnancy, inadequate GWG, and multiple births) that were associated or marginally associated with neurodevelopmental outcomes in *Aim 1 –single factor* analysis (**Table 4**).

**Table 4:** 5-y neurodevelopmental outcomes of SGA subgroups by co-occurrence of maternal smoking, inadequate GWG, and multiple births (N = 1050).

	Smoking	Inadequate GWG	Multiple births	N (%)	Adjusted mean difference in 5-y outcome (95% CI)^a^			
					Cognitive outcomes		Motor outcomes	
					Reading	Math	Gross	Fine
None	-	-	-	300 (29.5)	reference	reference	reference	reference
Single factor	+	-	-	100 (9.1)	0.01 (-3.07, 3.09)	0.12 (-2.10, 2.34)	0.31 (-0.10, 0.73)	0.18 (-0.17, 0.52)
	-	+	-	200 (21.2)	-1.37 (-3.73, 0.99)	-1.27 (-2.94, 0.39)	0.09 (-0.21, 0.38)	-0.16 (-0.43, 0.10)
	-	-	+	150 (16.0)	**-3.78 (-6.69, -0.88)**	**-2.15 (-4.08, -0.21)**	0.38 (0.03, 0.73)	-0.02 (-0.34, 0.30)
Two factors	+	+	-	<50 (5.7)	**-4.81 (-8.50, -1.12)**	**-2.95 (-5.51, -0.38)**	-0.12 (-0.61, 0.36)	-0.27 (-0.67, 0.13)
	+	-	+	<50 (2.9)	-5.12 (-11.39, 1.15)	**-4.00 (-7.92, -0.07)**	-0.13 (-0.93, 0.66)	**-0.68 (-1.28, -0.08)**
	-	+	+	150 (12.9)	**-6.32 (-9.25, -3.39)**	**-3.27 (-5.31, -1.22)**	0.28 (-0.13, 0.68)	-0.31 (-0.65, 0.03)
Three factors	+	+	+	<50 (2.6)	-4.45 (-10.03, 1.12)	-3.63 (-7.79, 0.54)	-0.18 (-1.08, 0.73)	0.27 (-0.35, 0.90)

GWG, gestational weight gain; CI, confidence interval.

Gross motor score range, 0–7; Fine motor score range, 0–5.

Significant results are bolded.

Definitions of prenatal risk factors:

Inadequate GWG: for singletons, total GWG less than 12.5 kg for underweight (pre-pregnancy BMI<18.5 kg/m2), 11.5 kg for normal weight (BMI, 18.5–24.9 kg/m2), 7 kg for overweight (BMI, 25–29.9 kg/m2), and 5 kg for obese women (BMI ≥30 kg/m2), respectively. For multiple births, total GWG less than 17 kg for underweight and normal weight, 14 kg for overweight, and 11 kg for obese women, respectively.

Multiple births: twins and triplets.

^a^ Adjusted for family socioeconomic status; maternal age at pregnancy, race/ethnicity, educational level, marital status, method of delivery, and diabetes during pregnancy; and child’s sex.

#### Aim 3 –Mediation

Based on the causal step method for medication analysis [34], we fitted 4 models with the same neurodevelopment outcome variable: the basic model (model 1) included SGA subgroups by the 3 co-occurring factors mentioned above and potential confounders, models 2 included the variables in model 1 and 5-minute Apgar score (**S1 Table**).

#### Supplemental analyses

We also ran several supplemental analyses to assess the robustness of our findings or to gain deeper insights on some research questions. First, in Aim 1, we compared neurodevelopmental outcomes between the etiological SGA subgroup with a specific risk factor to AGA without the corresponding risk factor (normal reference), which could help to assess the departure from normal neurodevelopment of healthy children (**S2 Table**). Second, we calculated Pearson correlations across reading, math, gross and fine motor scores at 5 y, which could help to assess their internal reliability (**S3 Table)**. Third, in Aim 2, we used the 4-category measure of maternal smoking to compare the 5-y neurodevelopmental outcomes across singleton SGA subgroups by co-occurrence of maternal smoking and inadequate GWG, which could provide insight on potential dose-response relationship **(S4 Table)**. Fourth, in order to explore the potential reasons for the differences in neurodevelopmental outcomes between multiple-birth SGA subgroups with and without ovulation stimulation, we compared their socio-demographics and parenting characteristics (**S5 Table)**.

## Results

### Sample characteristics

Compared with those in the excluded sample, mothers in the analytic sample were more educated, had higher family SES score, were more likely to have multiple births, and were less likely to smoke during pregnancy and be underweight; children in the analytic sample were less likely to be born preterm (**Table 1**).

### Neurodevelopmental outcomes of SGA

Children born SGA had lower mean reading (confounders-adjusted mean difference, -1.05 [95% confidence interval or CI, -1.93, -0.16]), math (-0.84 [-1.45, -0.22]), gross (-0.22 [-0.34, -0.10]) and fine (-0.22 [-0.32, -0.12]) motor scores than children born AGA (**Table 2**).

### Neurodevelopmental outcomes of SGA etiological subgroups by single factor (Aim 1)

Compared to the SGA subgroup with normal GWG, the SGA subgroup with inadequate GWG had lower mean reading (-2.26 [-3.98, -0.55]), math (-1.54 [-2.74, -0.35]) and fine motor (-0.19 [-0.38, 0.00]) scores (**Table 3**). Compared with the singleton SGA subgroup, the multiple-birth SGA subgroup had lower mean reading (-4.08 [-6.10, -2.06]) and math (-2.22 [-3.61, -0.84]) scores; these cognitive disadvantages were observed only among those without ovulation stimulation (reading, -4.50 [-6.64, -2.36]; math, -2.91 [-4.37, -1.44]) but not among those with ovulation stimulation (reading, -2.33 [-6.24, 1.57]; math, 0.63 [-1.86, 3.12]).

### Neurodevelopmental outcomes of SGA etiological subgroups by co-occurring factors (Aim 2)

We further stratified SGA by the co-occurrence of smoking during pregnancy, inadequate GWG, and multiple births, as they were associated or marginally associated with neurodevelopmental outcomes in single factor analysis shown above. Compared with the reference SGA subgroup without smoking, inadequate GWG, or multiple births, the multiple-birth SGA subgroup without smoking and inadequate GWG had lower mean reading (-3.78 [-6.69, -0.88]) and math (-2.15 [-4.08, -0.21]) score at 5 y (**Table 4**). The singleton SGA subgroup with both, but not single, of maternal smoking and inadequate GWG had lower mean reading (-4.81 [-8.50, -1.12]) and math (-2.95 [-5.51, -0.38]) scores. The SGA subgroup with co-occurrence of maternal smoking and multiple births had lower mean math (-4.00 [-7.92, -0.07]) and fine motor (-0.68 [-1.28, -0.08]) scores. The SGA subgroup with co-occurrence of maternal inadequate GWG and multiple births (12.9%) had lower mean reading (-6.32 [-9.25, -3.39]) and math (-3.27 [-5.31, -1.22]) scores.

In addition, among singleton SGA children, the co-occurrence of inadequate GWG and moderate smoking during pregnancy (vs. absence of these 2 risk factors) was associated with lower mean reading score (-4.55 [-8.06, -1.03]), while the co-occurrence of inadequate GWG and heavy smoking during pregnancy was associated with lower mean math score (-3.47 [-6.71, -0.22]) (**S4 Table**).

### Mediation analysis (Aim 3)

There was no significant difference in Apgar score across SGA subgroups by co-occurrence of maternal smoking, inadequate GWG, and multiple births (**S1 Table**). Therefore, Apgar score did not mediate the associations between SGA etiological subgroups and neurodevelopmental outcomes.

## Discussion

Within a large US prospective birth cohort, we used the etiological subgroup approach to examine the differences in neurodevelopmental outcomes at 5 y across subgroups of SGA based on each and co-occurrence of several important prenatal risk factors. We also examined the extent to which Apgar score could mediate the associations between SGA etiological subgroups and neurodevelopmental outcomes. Our results suggested that 1) the SGA subgroup with co-occurrence of maternal smoking and inadequate GWG was particularly disadvantaged in reading and math skills at 5 y; 2) the multiple-birth SGA subgroup without ovulation stimulation was at an increased risk of developmental delay in reading at 5 y; 3) these cognitive disadvantages could not be explained by low Apgar score at birth. Our novel findings supported the importance of considering prenatal factors in the understanding of heterogeneity in neurodevelopmental outcomes among SGA children. It is necessary to screen SGA children based on their potential etiology and identify SGA subgroups at the greatest neurodevelopmental risk for referrals to early intervention.

Consistent with the literature [6], we observed that on average children born SGA had worse cognitive and motor outcomes at 5 y than children born AGA, even after adjusting for a series of socio-demographic and pregnancy confounders. SGA is associated with comprised brain development and differentiation due to reduced oxygen and/or nutrient delivery [7].

One of our most important findings was that the singleton SGA subgroup with co-occurrence of maternal smoking during pregnancy and inadequate GWG had worse reading and math outcomes up to 5 y. This cognitive disadvantage seemed not be explained by low Apgar score. Prenatal exposure to tobacco metabolites may alter and damage fetal brain development reflected by small head circumference, lower cortical grey matter and total parenchymal volumes in the offspring [35], and thus increase risk of cognition delay, poor language skills, and behavioral problems in later life [28]. GWG is a proxy for maternal nutritional status or energy balance during pregnancy and placental function [10]. Malnutrition during pregnancy has been linked to fetal brain dysfunctions with widely distributed brain pathology [36]. Smoking pregnant women are at risk of inadequate GWG, possibly due to dietary restrictions related to the anorexic effects of tobacco [37]. The effects of co-occurrence of maternal smoking and inadequate GWG on the developing brain remain largely understudied. Based on the existing evidence on their separate effects, we propose several possible explanations for our novel finding: 1) the co-occurrence of maternal smoking and inadequate GWG acts as “double hits” to the fetal brain and causes more severe impairments than each factor separately. For example, for a pregnant women with inadequate GWG due to low caloric intake, smoking may further interfere with the efficiency of calorie utilization [38,39]; 2) fetal malnutrition due to maternal inadequate GWG may increase vulnerability of fetal brain to smoking-related hypoxia-ischemia and toxic tobacco products such as nicotine and carbon monoxide, and then lead to worse cognitive development; 3) although we adjusted for maternal education and other socio-demographics in this analysis, there must be some residual confounding by genetics [40], poor parenting and disadvantaged family environmental factors that put the child at high risk of cognitive delay.

Our second novel finding was that the multiple-birth SGA subgroup without ovulation stimulation was at an increased risk of developmental delay in reading. This finding is consistent with the literature on neurodevelopmental disadvantages related to multiple births [41]. It is hypothesized that multiple births are often restricted by crowded space and nutrition supply, and more likely to experience some severe fetal complications such as asphyxia and cerebral palsy; thus, have high risk of brain damage [42]. However, in our mediation analysis, the mean difference in reading score between singleton and multiple-birth SGA subgroups seemed not be explained by Apgar score. Alternatively, the disadvantaged postnatal environments such as twins’ competition for parental time and attention may help to explain this [43]. Interestingly, we did not observe adverse neurodevelopmental outcomes in the multiple-birth SGA subgroup with ovulation stimulation. As expected, our supplemental analysis showed that mothers of multiple-birth SGA subgroup with ovulation stimulation had more positive parenting at 2 and 4 y than those of multiple-birth SGA subgroup without ovulation stimulation (**S5 Table**). Positive parenting may well offset the potential disadvantages related to multiple births. Finally, we could not rule out the possibility that mother of multiple-birth SGA subgroup with ovulation stimulation might have involved genetic screening or selection of healthier embryos, which could be associated with the better neurodevelopmental outcomes.

We noticed that SGA children with co-occurrence of maternal smoking, inadequate GWG, and multiple births did not have statistically significantly worse neurodevelopmental outcomes than those without any of these 3 risk factors. This finding was different from our hypothesis (this subgroup being the worst). Particular caution is needed to interpret this finding, given the very small sample size of this SGA subgroup (2.6% within SGA). It might be related to survival bias of live babies, i.e., SGA newborns who survive in harsh intrauterine environment tend to be healthier than other babies who otherwise die before birth such as miscarriage.

We found SGA children as a whole group had poorer gross and fine motor development than AGA children, which was consistent with findings of some previous studies [44] but not with others [45]. However, we did not observe any substantial difference in motor development across SGA etiological subgroups. This null finding suggests that SGA per se predicts poor motor development in childhood, which does not depend on the potential cause(s) of SGA.

### Limitations

First, the attrition rate at 5-y visit was considerably high (~33%) in the ECLS-B sample. Our analytic sample was somewhat different from the excluded sample especially in maternal education, which could introduce selection bias. Second, the low prevalence of alcohol use limited statistical power to compare neurodevelopment across SGA subgroups by these factors. Also, we could not examine SGA subgroup due to some genetic factors such as maternal low birth weight as this information was unavailable in ECLS-B. Third, the relatively low reliability and validity of some non-medical measures on birth certificates needed to be taken in account when interpreting our findings [46]. For example, self-reported maternal smoking and alcohol use during pregnancy on birth certificates were subject to recall bias especially under-report since they are social undesirable behaviors [47]. Some pregnant women might not recall their pre-pregnancy weight accurately [48]. Fourth, we were unable to control for parental neurodevelopmental status such as IQ. But maternal education could, at least partially, reflect the level of maternal IQ. We could not explore other SGA-related perinatal complications as potential mediators due to insufficient power (e.g., meconium aspiration) or unavailable information (e.g., hypoglycemia and polycythemia).

## Conclusion

In summary, we found that multiple-birth SGA subgroup or singleton SGA subgroup with co-occurrence of smoking and inadequate GWG were particularly disadvantaged in cognitive development up to 5 y. From a clinical perspective, the effect size of these disadvantages appeared moderate (Cohen’s d<0.4) at individual level, however, a downward shift in mean neurodevelopmental level might result in many children with neurodevelopmental disabilities at population level [49]. If our novel findings can be replicated in other cohorts, further intervention research should assess if quitting smoking or avoiding inadequate GWG among smoking pregnant women could improve offspring’s neurodevelopmental outcomes. Also, we are concerned about the recent rapid increase in the prevalence of multiple births, as it seems to convey some cognitive disadvantages in the offspring.

## Supporting Information

S1 Table Differences in Apgar score across SGA subgroups by co-occurrence of maternal smoking, inadequate GWG, and multiple births (N = 1050). (DOCX)

S2 Table Neurodevelopmental outcomes at 5 y of SGA vs AGA subgroups by single prenatal risk factor (N = 5300). (DOC)

S3 Table Pearson correlations between neurodevelopmental outcomes at 5 y (N = 5300). (DOC)

S4 Table 5-y neurodevelopmental outcomes of singleton SGA subgroups by co-occurrence of maternal smoking and inadequate GWG (N = 700). (DOCX)

S5 Table Comparisons of socio-demographics and parenting characteristics between singleton, multiple-birth SGA subgroups with and without ovulation stimulation (N = 1050). (DOCX)

## Abbreviations

AGA: appropriate-for-gestational-age
BMI: body mass index
CI: confidence interval
GWG: gestational weight gain
SGA: small-for-gestational-age
ECLS-B: Early Childhood Longitudinal Study-Birth Cohort
LMP: last menstrual period
