# Supplementary material for: Etiological Subgroups of Small-for-Gestational-Age: Differential Neurodevelopmental Outcomes
Source: PLoS One. 2016 Aug 8;11(8):e0160677. doi: 10.1371/journal.pone.0160677 (PMC4976943; doi:10.1371/journal.pone.0160677)
Supplement: S1 Table — (DOCX) [file pone.0160677.s001.docx]

**S1 Table. Differences in Apgar score across SGA subgroups by co-occurrence of maternal smoking, inadequate GWG, and multiple births (N=1050)**

|  | **Smoking** | **Inadequate GWG** | **Multiple births** | **N (%)** | **Mean difference in Apgar score (95% CI)^a^** |
| --- | --- | --- | --- | --- | --- |
| None | - | - | - | 300 (29.5) | Reference |
| Single factor | + | - | - | 100 (9.1) | -0.01 (-0.26, 0.25) |
|  | - | + | - | 200 (21.2) | -0.19 (-0.39, 0.00) |
|  | - | - | + | 150 (16.0) | 0.06 (-0.16, 0.28) |
| Two factors | + | + | - | 50 (5.7) | 0.11 (-0.20, 0.43) |
|  | + | - | + | <50 (2.9) | 0.23 (-0.01, 0.47) |
|  | - | + | + | 150 (12.9) | 0.10 (-0.10, 0.31) |
| Three factors | + | + | + | <50 (2.6) | 0.15 (-0.12, 0.43) |

GWG, gestational weight gain; CI, confidence interval.

Significant results are bolded.

Definitions of prenatal risk factors:

Inadequate GWG: for singletons, total GWG less than 12.5 kg for underweight (pre-pregnancy BMI<18.5 kg/m2), 11.5 kg for normal weight (BMI, 18.5-24.9 kg/m2), 7 kg for overweight (BMI, 25-29.9 kg/m2), and 5 kg for obese women (BMI ≥30 kg/m2), respectively. For multiple births, total GWG less than 17 kg for underweight and normal weight, 14 kg for overweight, and 11 kg for obese women, respectively.

Multiple births: twins and triplets.

^a^ Adjusted for family socioeconomic status; maternal age at pregnancy, race/ethnicity, educational level, marital status, method of delivery, and diabetes during pregnancy; and child’s sex
