# Supplementary material for: Etiological Subgroups of Small-for-Gestational-Age: Differential Neurodevelopmental Outcomes
Source: PLoS One. 2016 Aug 8;11(8):e0160677. doi: 10.1371/journal.pone.0160677 (PMC4976943; doi:10.1371/journal.pone.0160677)
Supplement: S2 Table — (DOC) [file pone.0160677.s002.doc]

**S2 Table. Neurodevelopmental outcomes at 5 y of SGA vs AGA subgroups by single prenatal risk factor (N=5300)**

|  |  | **Adjusted mean difference in 5-y cognitive outcome (95% CI)^a^** | | | | |
| --- | --- | --- | --- | --- | --- | --- |
|  |  | **Cognition** | |  | **Motor** | |
|  | **N (%)** | **Reading** | **Math** |  | **Gross** | **Fine** |
| **By maternal pre-pregnancy underweight** |  |  |  |  |  |  |
| AGA without underweight | 4000 (75.1) | Reference | Reference |  | Reference | Reference |
| AGA with underweight | 250 (4.9) | -0.03 (-1.83, 1.77) | 0.59 (-0.66, 1.84) |  | -0.13 (-0.35, 0.08) | 0.13 (-0.03, 0.29) |
| SGA without underweight | 1000 (18.6) | **-1.08 (-2.00, -0.16)** | **-0.86 (-1.50, -0.23)** |  | **-0.22 (-0.34, -0.10)** | **-0.22 (-0.33, -0.11)** |
| SGA with underweight | 100 (1.5) | -0.58 (-3.47, 2.30) | 0.02 (-2.00, 2.03) |  | **-0.36 (-0.68, -0.03)** | -0.10 (-0.38, 0.19) |
| **By maternal short stature** |  |  |  |  |  |  |
| AGA without short stature | 3150 (59.5) | Reference | Reference |  | Reference | Reference |
| AGA with short stature | 1100 (20.5) | -0.18 (-1.18, 0.82) | 0.20 (-0.49, 0.89) |  | -0.01 (-0.13, 0.10) | 0.00 (-0.10, 0.10) |
| SGA without short stature | 700 (13.3) | **-1.07 (-2.13, -0.02)** | **-0.76 (-1.49, -0.03)** |  | **-0.33 (-0.48, -0.19)** | **-0.24 (-0.36, -0.11)** |
| SGA with short stature | 350 (6.7) | -1.12 (-2.63, 0.38) | -0.85 (-1.89, 0.20) |  | -0.01 (-0.19, 0.18) | **-0.18 (-0.35, -0.02)** |
| **By maternal smoking during pregnancy** |  |  |  |  |  |  |
| AGA without smoking | 3350 (63.7) | Reference | Reference |  | Reference | Reference |
| AGA with quitted smoking | 400 (7.4) | -0.33 (-1.80, 1.14) | -0.11 (-1.10, 0.89) |  | 0.00 (-0.18, 0.18) | -0.06 (-0.21, 0.10) |
| AGA with moderate smoking | 250 (4.4) | **-2.35 (-4.18, -0.52)** | **-1.61 (-2.93, -0.28)** |  | -0.14 (-0.38, 0.10) | **-0.23 (-0.44, -0.02)** |
| AGA with heavy smoking | 250 (4.5) | -1.41 (-3.36, 0.54) | -1.38 (-2.82, 0.07) |  | **-0.32 (-0.56, -0.07)** | **-0.24 (-0.45, -0.04)** |
| SGA without smoking | 750 (14.4) | **-1.18 (-2.22, -0.14)** | **-0.85 (-1.56, -0.14)** |  | **-0.23 (-0.37, -0.10)** | **-0.21 (-0.33, -0.09)** |
| SGA with quitted smoking | 100 (1.6) | -1.61 (-4.30, 1.08) | -1.25 (-3.23, 0.72) |  | -0.15 (-0.56, 0.26) | -0.35 (-0.74, 0.03) |
| SGA with moderate smoking | 100 (1.9) | -1.27 (-3.51, 0.97) | -0.85 (-2.64, 0.94) |  | -0.15 (-0.48, 0.19) | **-0.31 (-0.60, -0.02)** |
| SGA with heavy smoking | 100 (2.1) | -1.91 (-4.81, 0.98) | -2.29 (-4.22, -0.36) |  | -0.53 (-0.90, -0.16) | **-0.45 (-0.74, -0.16)** |
| **By maternal alcohol use during pregnancy** |  |  |  |  |  |  |
| AGA without alcohol use | 4100 (77.4) | Reference | Reference |  | Reference | Reference |
| AGA with alcohol use | 150 (2.6) | -1.63 (-3.76, 0.50) | -0.69 (-2.20, 0.82) |  | 0.21 (-0.07, 0.48) | -0.06 (-0.30, 0.17) |
| SGA without alcohol use | 1000 (19.1) | **-1.07 (-1.98, -0.15)** | **-0.89 (-1.52, -0.26)** |  | **-0.23 (-0.35, -0.11)** | **-0.23 (-0.34, -0.13)** |
| SGA with alcohol use | 50 (1.0) | -1.90 (-5.29, 1.49) | -0.30 (-2.57, 1.97) |  | 0.05 (-0.36, 0.47) | -0.03 (-0.48, 0.43) |
| **By maternal GWG** |  |  |  |  |  |  |
| AGA with normal GWG | 2950 (55.5) | Reference | Reference |  | Reference | Reference |
| AGA with inadequate GWG | 1300 (24.5) | **-1.29 (-2.22, -0.36)** | **-1.28 (-1.91, -0.65)** |  | **-0.15 (-0.26, -0.04)** | **-0.18 (-0.27, -0.09)** |
| SGA with normal GWG | 600 (11.5) | -0.56 (-1.68, 0.57) | -0.66 (-1.44, 0.11) |  | **-0.24 (-0.39, -0.09)** | **-0.20 (-0.33, -0.07)** |
| SGA with inadequate GWG | 450 (8.5) | **-2.76 (-4.11, -1.42)** | **-2.09 (-3.03, -1.15)** |  | **-0.30 (-0.48, -0.13)** | **-0.39 (-0.54, -0.23)** |
| **By maternal hypertensive conditions** |  |  |  |  |  |  |
| AGA without hypertensive conditions | 3950 (74.8) | Reference | Reference |  | Reference | Reference |
| AGA with hypertensive conditions | 250 (5.2) | -0.08 (-1.82, 1.65) | -0.13 (-1.37, 1.11) |  | -0.22 (-0.45, 0.01) | -0.18 (-0.36, 0.00) |
| SGA without hypertensive conditions | 950 (17.6) | **-1.10 (-2.04, -0.16)** | **-0.90 (-1.54, -0.25)** |  | **-0.21 (-0.33, -0.09)** | **-0.23 (-0.33, -0.12)** |
| SGA with hypertensive conditions | 150 (2.4) | -0.72 (-3.06, 1.63) | -0.48 (-2.11, 1.16) |  | **-0.43 (-0.75, -0.10)** | **-0.30 (-0.58, -0.02)** |
| **By multiple births** |  |  |  |  |  |  |
| Singleton AGA | 3550 (67.1) | Reference | Reference |  | Reference | Reference |
| Multiple-birth AGA | 700 (12.8) | **-2.80 (-4.13, -1.46)** | **-2.69 (-3.60, -1.78)** |  | **-0.21 (-0.38, -0.04)** | **-0.29 (-0.42, -0.15)** |
| Without ovulation stimulation | 550 (10.1) | **-3.46 (-4.93, -1.99)** | **-3.16 (-4.17, -2.15)** |  | **-0.22 (-0.41, -0.04)** | **-0.33 (-0.47, -0.18)** |
| With ovulation stimulation | 150 (2.8) | -0.39 (-2.94, 2.17) | -0.90 (-2.63, 0.82) |  | -0.15 (-0.47, 0.16) | -0.15 (-0.41, 0.11) |
| Singleton SGA | 700 (13.1) | -0.31 (-1.42, 0.81) | **-0.80 (-1.57, -0.02)** |  | **-0.30 (-0.44, -0.16)** | **-0.25 (-0.37, -0.12)** |
| Multiple-birth SGA | 350 (6.9) | **-4.33 (-5.86, -2.79)** | **-2.99 (-4.03, -1.95)** |  | **-0.21 (-0.42, 0.00)** | **-0.35 (-0.54, -0.17)** |
| Without ovulation stimulation | 300 (5.4) | **-4.62 (-6.26, -2.99)** | **-3.59 (-4.74, -2.45)** |  | **-0.20 (-0.43, 0.02)** | **-0.38 (-0.59, -0.17)** |
| With ovulation stimulation | 50 (1.5) | -3.07 (-6.49, 0.36) | -0.76 (-2.84, 1.31) |  | -0.21 (-0.66, 0.24) | -0.25 (-0.58, 0.08) |

Multiple births: twins and triplets

SGA, small-for-gestational-age; AGA, appropriate-for-gestational-age; GWG, gestational weight gain; CI, confidence interval.

Gross motor score range, 0-7; Fine motor score range, 0-5.

Significant results are bolded.

Definitions of prenatal risk factors:

Pre-pregnancy underweight: BMI<18.5kg/m2;

Maternal short stature: height ≤157.5cm;

Smoking: never smoking, quitted smoking (smoking before pregnancy, but not during pregnancy), moderate smoking (1-9 cigarettes/day during pregnancy) and heavy smoking (≥10 cigarettes/day during pregnancy)

Inadequate GWG: for singletons, total GWG less than 12.5 kg for underweight (pre-pregnancy BMI<18.5 kg/m2), 11.5 kg for normal weight (BMI, 18.5-24.9 kg/m2), 7 kg for overweight (BMI, 25-29.9 kg/m2), and 5 kg for obese women (BMI ≥30 kg/m2), respectively. For multiple births, total GWG less than 17 kg for underweight and normal weight, 14 kg for overweight, and 11 kg for obese women, respectively.

Hypertensive conditions: chronic hypertension, gestational hypertension, preeclampsia, and eclampsia.

^a^ Adjusted for family socioeconomic status; maternal age at pregnancy, race/ethnicity, educational level, marital status, method of delivery, and diabetes during pregnancy; and child’s sex.
