# Supplementary material for: Etiological Subgroups of Small-for-Gestational-Age: Differential Neurodevelopmental Outcomes
Source: PLoS One. 2016 Aug 8;11(8):e0160677. doi: 10.1371/journal.pone.0160677 (PMC4976943; doi:10.1371/journal.pone.0160677)
Supplement: S3 Table — (DOC) [file pone.0160677.s003.doc]

**S3 Table. Pearson correlations^a^ between neurodevelopmental outcomes at 5 y (N=5300)**

|  | **Reading** | **Math** | **Gross motor** |
| --- | --- | --- | --- |
| Reading | 1.00 |  |  |
| Math | **0.82** | 1.00 |  |
| Gross motor | **0.29** | **0.35** | 1.00 |
| Fine motor | **0.50** | **0.55** | **0.34** |

^a^ All P-values<0.05.
