# Supplementary material for: Etiological Subgroups of Small-for-Gestational-Age: Differential Neurodevelopmental Outcomes
Source: PLoS One. 2016 Aug 8;11(8):e0160677. doi: 10.1371/journal.pone.0160677 (PMC4976943; doi:10.1371/journal.pone.0160677)
Supplement: S4 Table — (DOCX) [file pone.0160677.s004.docx]

**S4 Table. 5-y neurodevelopmental outcomes of singleton SGA subgroups by co-occurrence of maternal smoking and inadequate GWG (N=700)**

|  |  |  | **Adjusted mean difference in 5-y outcome (95% CI)^a^** | | | | | | |
| --- | --- | --- | --- | --- | --- | --- | --- | --- | --- |
| **Smoking** | **Inadequate GWG** |  | **Cognitive outcomes** | | |  | **Motor outcomes** | | |
|  |  | **N (%)** | **Reading** |  | **Math** |  | **Gross motor** |  | **Fine motor** |
| None | - | 300 (40.0) | Reference |  | Reference |  | Reference |  | Reference |
| Quitted | - | <50 (5.3) | 0.48 (-3.92, 4.88) |  | -0.53 (-3.66, 2.59) |  | 0.14 (-0.42, 0.71) |  | -0.08 (-0.59, 0.43) |
| Moderate (1-9) | - | 50 (7.5) | 0.92 (-2.37, 4.21) |  | 0.39 (-2.10, 2.88) |  | 0.42 (-0.04, 0.87) |  | 0.03 (-0.37, 0.43) |
| Heavy (10+) | - | <50 (6.2) | -1.29 (-5.39, 2.81) |  | -1.05 (-3.88, 1.78) |  | -0.17 (-0.70, 0.36) |  | -0.14 (-0.57, 0.28) |
| Never | + | 200 (29.6) | -1.67 (-3.73, 0.38) |  | -1.24 (-2.67, 0.19) |  | 0.04 (-0.22, 0.29) |  | -0.22 (-0.45, 0.00) |
| Quitted | + | <50 (2.7) | -3.49 (-8.25, 1.27) |  | -2.63 (-5.96, 0.70) |  | 0.20 (-0.48, 0.88) |  | -0.22 (-0.85, 0.42) |
| Moderate (1-9) | + | <50 (3.7) | -**4.55 (-8.06, -1.03)** |  | -1.76 (-4.37, 0.84) |  | -0.20 (-0.76, 0.35) |  | -0.13 (-0.57, 0.31) |
| Heavy (10+) | + | <50 (5.0) | -3.07 (-7.67, 1.52) |  | **-3.47 (-6.71, -0.22)** |  | -0.21 (-0.81, 0.40) |  | -0.18 (-0.66, 0.31) |

GWG, gestational weight gain; CI, confidence interval.

Gross motor score range, 0-7; Fine motor score range, 0-5.

Significant results are bolded.

Definitions of prenatal risk factors:

Smoking: never smoking, quitted smoking (smoking before pregnancy, but not during pregnancy), moderate smoking (1-9 cigarettes/day during pregnancy) and heavy smoking (≥10 cigarettes/day during pregnancy)

Inadequate GWG: for singletons, total GWG less than 12.5 kg for underweight (pre-pregnancy BMI<18.5 kg/m2), 11.5 kg for normal weight (BMI, 18.5-24.9 kg/m2), 7 kg for overweight (BMI, 25-29.9 kg/m2), and 5 kg for obese women (BMI ≥30 kg/m2), respectively. For multiple births, total GWG less than 17 kg for underweight and normal weight, 14 kg for overweight, and 11 kg for obese women, respectively.

^a^ Adjusted for family socioeconomic status; maternal age at pregnancy, race/ethnicity, educational level, marital status, method of delivery, and diabetes during pregnancy; and child’s sex
