# Supplementary material for: Etiological Subgroups of Small-for-Gestational-Age: Differential Neurodevelopmental Outcomes
Source: PLoS One. 2016 Aug 8;11(8):e0160677. doi: 10.1371/journal.pone.0160677 (PMC4976943; doi:10.1371/journal.pone.0160677)
Supplement: S5 Table — (DOCX) [file pone.0160677.s005.docx]

**S5 Table. Comparisons of socio-demographics and parenting characteristics between singleton, multiple-birth SGA subgroups with and without ovulation stimulation (N=1050).**

|  | **Singleton SGA (N=700)**  **(1)** | |  | **Multiple-birth SGA without ovulation stimulation (N=250)**  **(2)** | |  | **Multiple-birth SGA with ovulation stimulation (N=100)**  **(3)** | | **Pairwise comparison *P-value^a^*** | | |
| --- | --- | --- | --- | --- | --- | --- | --- | --- | --- | --- | --- |
|  |  |  |  |  |  |  |  |  | **1 vs. 2** | **1 vs. 3** | **2 vs. 3** |
| **Characteristics** | **%** | **Mean (SD)** |  | **%** | **Mean (SD)** |  | **%** | **Mean (SD)** |  |  |  |
| **HOUSEHOLD** |  |  |  |  |  |  |  |  |  |  |  |
| Family socioeconomic status |  | -0.2 (0.9) |  |  | 0.0 (0.9) |  |  | 0.8 (0.6) | **<0.001** | **<0.001** | **<0.001** |
| **MOTHER** |  |  |  |  |  |  |  |  |  |  |  |
| Age at pregnancy |  | 26.4 (6.4) |  |  | 27.9 (6.4) |  |  | 33.0 (4.8) | **0.002** | **<0.001** | **<0.001** |
| Race |  |  |  |  |  |  |  |  |  |  |  |
| Non-Hispanic white | 38.9 |  |  | 61.1 |  |  | 90.1 |  |  |  |  |
| Non-Hispanic black | 26.5 |  |  | 22.1 |  |  | 1.2 |  |  |  |  |
| Hispanics | 15.4 |  |  | 14.7 |  |  | 6.2 |  | **<0.001** | **<0.001** | **<0.001** |
| Asian/Pacific Islander | 13.9 |  |  | 1.4 |  |  | 2.5 |  |  |  |  |
| American Indian | 5.3 |  |  | 0.7 |  |  | 0.0 |  |  |  |  |
| Education level |  |  |  |  |  |  |  |  |  |  |  |
| Below high school | 23.1 |  |  | 17.5 |  |  | 12.4 |  |  |  |  |
| High school or equivalent | 35.3 |  |  | 28.1 |  |  | 54.3 |  | **0.003** | **<0.001** | **<0.001** |
| College | 33.4 |  |  | 45.3 |  |  | 33.3 |  |  |  |  |
| Graduate or above | 8.2 |  |  | 9.1 |  |  | 0.0 |  |  |  |  |
| Married | 58.0 |  |  | 64.6 |  |  | 98.8 |  | 0.056 | **<0.001** | **<0.001** |
| Vaginal delivery | 67.9 |  |  | 34.4 |  |  | 32.1 |  | **<0.001** | **<0.001** | 0.701 |
| Diabetes during pregnancy | 5.0 |  |  | 0.0 |  |  | 4.9 |  | **<0.001** | 0.974 | **<0.001** |
| **CHILD** |  |  |  |  |  |  |  |  |  |  |  |
| Gestational age, weeks |  | 37.9 (3.1) |  |  | 36.9 (2.2) |  |  | 36.7 (2.9) | **<0.001** | **0.001** | 0.420 |
| Parenting at 2 y |  |  |  |  |  |  |  |  |  |  |  |
| Parental sensitivity |  | 4.6 (1.0) |  |  | 4.6 (1.0) |  |  | 5.3 (0.9) | 0.860 | **<0.001** | **<0.001** |
| Parental positive regard |  | 4.2 (1.0) |  |  | 4.2 (1.1) |  |  | 4.8 (1.0) | 0.735 | **<0.001** | **<0.001** |
| Parental negative regard |  | 1.2 (0.6) |  |  | 1.1 (0.5) |  |  | 1.0 (0.2) | 0.355 | **<0.001** | **0.016** |
| Parental intrusiveness |  | 1.2 (0.6) |  |  | 1.2 (0.5) |  |  | 1.1 (0.4) | 0.063 | **0.006** | 0.272 |
| Parental cognitive  development stimulation |  | 4.0 (1.1) |  |  | 4.0 (0.9) |  |  | 4.5 (1.2) | 0.855 | **0.001** | **<0.001** |
| Parental detachment |  | 1.1 (0.5) |  |  | 1.2 (0.6) |  |  | 1.0 (0.0) | 0.141 | **<0.001** | **0.001** |
| Parenting at 4 y |  |  |  |  |  |  |  |  |  |  |  |
| Parental emotion support |  | 4.3 (0.9) |  |  | 4.5 (0.9) |  |  | 4.8 (0.8) | **0.006** | **<0.001** | **0.014** |
| Parental negative regard |  | 1.3 (0.6) |  |  | 1.2 (0.4) |  |  | 1.1 (0.2) | **0.005** | **<0.001** | **0.003** |
| Parental intrusiveness |  | 1.6 (0.9) |  |  | 1.5 (0.8) |  |  | 1.4 (0.7) | **0.042** | **0.003** | 0.120 |
| Parental cognitive  development stimulation |  | 4.2 (1.0) |  |  | 4.2 (1.0) |  |  | 4.6 (0.7) | 0.269 | **0.001** | **0.006** |
| Parental detachment |  | 1.4 (0.8) |  |  | 1.4 (0.7) |  |  | 1.3 (0.5) | 0.901 | 0.064 | 0.111 |

SD, standard deviation; SGA, small-for-gestational-age.
^a^ Chi-square test for categorical variables and *t*-test for continuous variables.

Significant results are bolded.

**Parenting measure methods**. In ECLS-B, parenting was assessed by the Two Bags Task at the child’s ages of 2 y and 4 y. The Two Bags Task (10 minutes) is a simplified version of the Three Bags Task (15 minutes) used in large-scale studies. Briefly, the parent-child dyad was instructed to play with the bags in numerical order for 10 minutes. The parent and child were videotaped while engaged in the activity. These videotapes were coded by trained observers .The trained coder reviewed the videotape on parent-child interaction, and then rated each parenting domain on a 7-point Likert scale ranging from very low (1) to very high (7). The 2-y parenting scale had 6 subscales (domains): parental sensitivity, positive regard, stimulation of cognitive development, intrusiveness, negative regard, and detachment. The 4-y parenting assessment had 5 subscales: parental emotional supportiveness (replacing sensitivity and positive regard at 2 y), stimulation of cognitive development, intrusiveness, negative regard, and detachment.
